# MicroRNA-17-5p regulated apoptosis-related protein expression and radiosensitivity in oral squamous cell carcinoma caused by betel nut chewing

**DOI:** 10.18632/oncotarget.9856

**Published:** 2016-06-06

**Authors:** Szu-Yuan Wu, Alexander T.H. Wu, Shing-Hwa Liu

**Affiliations:** ^1^ Institute of Toxicology, College of Medicine, National Taiwan University, Taipei, Taiwan; ^2^ Department of Radiation Oncology, Wan Fang Hospital, Taipei Medical University, Taipei, Taiwan; ^3^ Department of Internal Medicine, School of Medicine, College of Medicine, Taipei Medical University, Taipei, Taiwan; ^4^ Department of Biotechnology, Hungkuang University, Taichung, Taiwan; ^5^ The Ph.D. Program for Translational Medicine, Taipei Medical University, Taipei, Taiwan; ^6^ Department of Pediatrics, College of Medicine, National Taiwan University, Taipei, Taiwan; ^7^ Department of Medical Research, China Medical University Hospital, China Medical University, Taichung, Taiwan

**Keywords:** miR-17-5p, OC3 cells, p53, radiation, apoptosis

## Abstract

Betel nut chewing is associated with oral cavity cancer. Radiotherapy is one of the therapeutic approaches. Here, we used miR-17-5p antisense oligonucleotides (AS-ODNs) and human apoptosis protein array to clarify which apoptosis-related proteins are increased or decreased by miR-17-5p in betel nut chewing- oral squamous cell carcinoma OC3 cells. Furthermore, miR-17-5p AS-ODN was used to evaluate the radio-sensitization effects both *in vitro* and *in vivo*. An OC3 xenograft tumor model in severe combined immunodeficiency mice was used to determine the effect of miR-17-5p AS ODN on tumor irradiation. We simultaneously detected the relative expressions of 35 apoptosis-related proteins in irradiated OC3 cells that were treated with miR-17-5p AS-ODN or a control ODN. Several proteins, including p21, p53, TNF RI, FADD, cIAP-1, HIF-1α, and TRAIL R1, were found to be up- or downregulated by miR-17-5p in OC3 cells; their expression patterns were also confirmed by Western blotting. We further clarified the role of p53 in irradiated OC3 cells, using a p53 overexpression strategy. The results revealed that the enhancement of p53 expression significantly enhanced radiation-induced G2/M arrest of the OC3 cells. In the *in vivo* study, treatment of miR-17-5p AS-ODN before irradiation significantly enhanced p53 expression and reduced tumor growth. These results suggest that miR-17-5p increases or decreases apoptosis-related proteins in irradiated OC3 cells; its effect on p53 protein expression contributes to the modulation of the radiosensitivity of the OC3 cells.

## INTRODUCTION

Head and neck cancer is the sixth most common cancer worldwide, with approximately 650000 cases and 200000 deaths annually. In the United States, approximately 54000 new head and neck cancer cases are annually diagnosed [1]. In certain Asian countries, such as Taiwan, head and neck cancer is the fourth leading cause of cancer deaths and the sixth most common cancer [2]. In Taiwan, more than 99% of head and neck cancers are squamous cell carcinomas, and more than 88% of patients with head and neck cancer have a betel nut-chewing habit [3, 4]. Betel nut chewers have higher incidences of local recurrence, distant metastasis, and secondary primary cancers as well as poorer disease-specific and overall survival than do non-chewers [3]. Most Taiwanese patients with head and neck cancer are males with a betel nut-chewing habit and a median age of 53 years, who may be a crucial economic contributor to their families [2, 5, 6]. Locoregional diseases (stages III–IV) are amenable to multimodality therapy–typically a combination of chemotherapy and radiotherapy (RT). However, the 5-year survival rate after multimodality therapy remains low (<50%) [7]; therefore, effective treatments and useful radiation enhancers are required.

Betel (*Areca catechu*) nut chewing is associated with the development of several types of oral cancers [8]. Betel nut chewing, which is widespread in Taiwan, is an independent risk factor for the development of squamous cell head and neck cancer. The effects appear to be synergistic with tobacco and alcohol [9]. RT is one of the therapeutic approaches for oral cancer. However, the molecular radiobiology of betel nut chewing-related cancer cells remains unclear. The mechanism underlying radiation-induced cell death includes the induction of apoptosis, and recent studies have revealed that micro (mi)RNAs (miRs) may post-transcriptionally increase or decrease the expression of stress response genes that control cell survival and apoptosis. MiRs are small (−22 nucleotides) regulatory RNAs that play crucial roles in various normal and pathological processes. Their dysregulation was implicated in the pathogenesis of various diseases [10].

MiR-17-92 (miR-17) is a polycistronic miR that encodes seven mature miRs and was the first miR characterized to be associated with cancer [11–13]. MiR-19, which is a component of the miR-17/Oncomir-1 miR polycistron, interferes with the expression of the antiapoptotic Ras homolog B (rhoB). This process also requires the binding of the human antigen R protein, an AU-rich element-binding protein, to the 3′-untranslated region of the rhoB messenger (m)RNA in keratinocytes exposed to ultraviolet radiation [14]. That study also revealed that translation inhibition mediated by the miR-17/Oncomir-1 miR polycistron is associated with an apoptotic response. The role of the miR-17 polycistron in response to RT in oral cancer remains unclear. Therefore, we examined radiation-induced changes in miR-17 expression and its function in oral carcinoma 3 (OC3) cells, an oral carcinoma cell line that was established from a 57-year-old Taiwanese patient having oral squamous cell carcinoma; this patient was a long-term betel nut chewer who did not smoke [15]. Our previous study revealed that miR-17-5p, a miR-17-92 polycistronic miR, is enhanced in the irradiated OC3 cancer cell line, and miR-17-5p was also observed to inhibit the downstream p21 expression and reduce radiosensitivity [16]. In this study, we used microRNA-17-5p (miR-17-5p) antisense (AS) oligonucleotides (ODN) and a human apoptosis protein array to identify apoptosis-related proteins that are increased or decreased by miR-17-5p.

## RESULTS

### MicroRNA-17-5p-regulated apoptosis-related protein expression in irradiated OC3 cells

**Figure 1:**
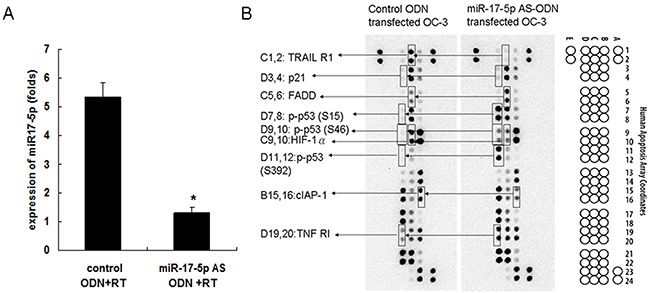
Effects of miR-17-5p AS ODN on the expression of OC3 cell apoptosis-related proteins The OC3 cells were pretreated with miR-17-5p AS ODN or control ODN for 48 h, followed by irradiation with 5 Gy. **A.** Six hours later, the expression of miR-17-5p was determined through real-time PCR. The miR expression was normalized to sno202 RNA and is presented in arbitrary units. Data from the miR-17 AS ODN and control ODN groups were compared. *P < 0.05. **B.** After 24 h, the total cell lysates were collected for the apoptosis protein array analysis. Data shown are representative images of three independent experiments; significantly changed protein spots (as revealed in Figure 2) are indicated.

Our previous study revealed that miR-17-5p could be induced in irradiated OC3 cells [16]. To determine the effects of miR-17-5p on OC3 cell apoptosis related-protein expression, the OC3 cells were pretreated with the miR-17-5p AS ODN or control ODN for 48 h before irradiation with 5 Gy. Furthermore, the inhibition effect of miR-17-5p AS ODN on irradiation-induced miR-17-5p expression in the OC3 cells was verified through real-time PCR (Figure 1A). The results revealed that irradiation-induced miR-17-5p expression was significantly inhibited by miR-17-5p AS ODN but not by control ODN in irradiated OC3 cells. The expression level of miR-17-5p was 5.34 ± 0.51 folds in the control ODN group and 1.3 ± 0.2 folds in the miR-17-5p AS ODN group (*P* < 0.05). Furthermore, the total cell lysates were collected for the apoptosis protein analysis. By using the human apoptosis protein array, we simultaneously detected the relative expression of 35 apoptosis-related proteins in irradiated OC3 cells. Images of the apoptosis array (Figure 1) revealed that apoptosis-related proteins, namely p21, p53 (phosphorylated at S15, S46, or S392), TNF RI, FADD, cIAP-1, HIF-1α, and TRAIL R1, exhibited different expression levels in the irradiated OC3 cells pretreated with miR-17-5p AS ODN and the cells treated with control ODN (Figure 1B).

### MicroRNA-17-5p-downregulated the apoptotic proteins of p21, p53, TNF RI, and FADD, and upregulated the apoptotic proteins of cIAP-1, HIF-1α, and TRAIL R1 in irradiated OC3 cells

**Figure 2:**
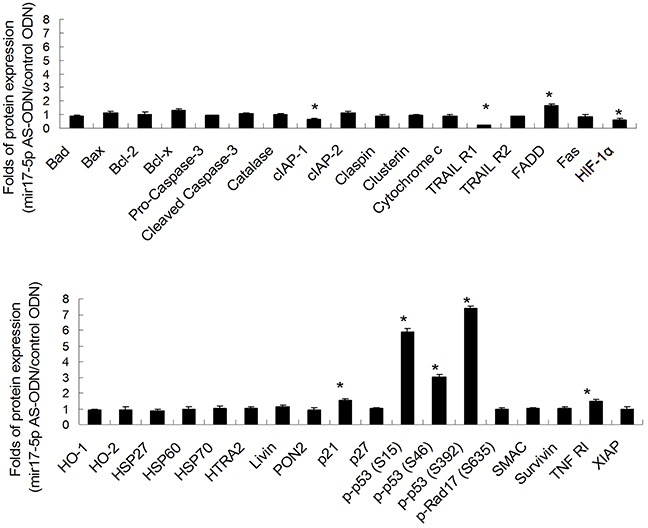
Quantitative results of apoptosis protein arrays from miR-17-5p AS ODN-treated OC3 cells Quantitative results of apoptosis protein arrays, as described in Figure 1. The figure shows the ratios of the miR-17-5p AS ODN treatment versus the control ODN treatment (n = 3). *P > 1.5 was considered significant.

**Figure 3:**
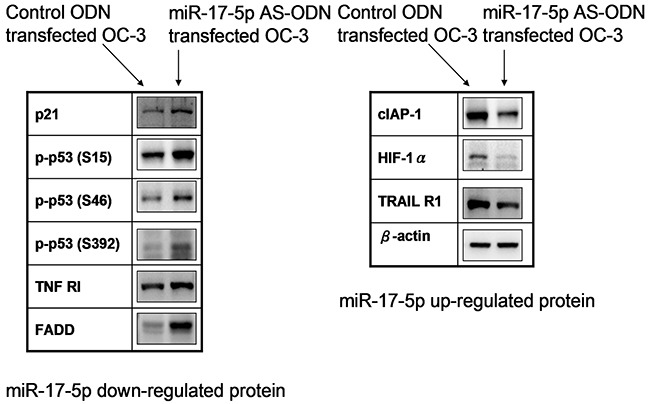
MiR-17-5p-regulated protein expressions in OC3 cells The OC3 cells were pretreated with the miR-17-5p AS ODN or control ODN for 48 h. After 24 h, the total cell lysates were collected for Western blotting. Data shown are representative images of three independent experiments. MiR-17-5p-downregulated (left panel) or -upregulated (right panel) proteins are indicated.

Quantitative results of the apoptosis array revealed that miR-17-5p downregulated the expression of p21, p53, TNF RI, and FADD. However, miR-17-5p upregulated the expressions of cIAP-1, HIF-1α, and TRAIL R1. The results are presented as ratios of miR-17-5p AS ODN treatment versus the control ODN treatment; we defined ratios of >1.5 or <0.6 as a significant change. The ratios were 1.54 ± 0.12, 5.9 ± 0.21, and 3.04 ± 0.13 for p21, p-p53-S15, and p-p53-S46, respectively. Furthermore, for p-p53-S392, TNF RI, and FADD, the ratios were 7.37 ± 0.14, 1.5 ± 0.12, and 1.69 ± 0.13, respectively. The ratios were 0.6 ± 0.04 for cIAP-1, 0.59 ± 0.13 for HIF-1α, and 0.21 ± 0.02 for TRAIL R1 (Figure 2). We further confirmed the expression of p21, p-p53 (phosphorylated at S15, S46, and S392), TNF RI, FADD, cIAP-1, HIF-1α, and TRAIL R1 in irradiated OC3 cells through Western blotting (Figure 3). The results revealed that the downregulated apoptotic patterns of p21, p53, TNF RI, and FADD (Figure 3, left panel) and upregulated apoptotic patterns of cIAP-1, HIF-1α, and TRAIL R1 (Figure 3, right panel) were similar in both groups analyzed using apoptosis array and Western blotting.

### Enhanced radiation-induced G2/M phase arrest in OC3 cells by overexpression of the p53 protein

**Figure 4:**
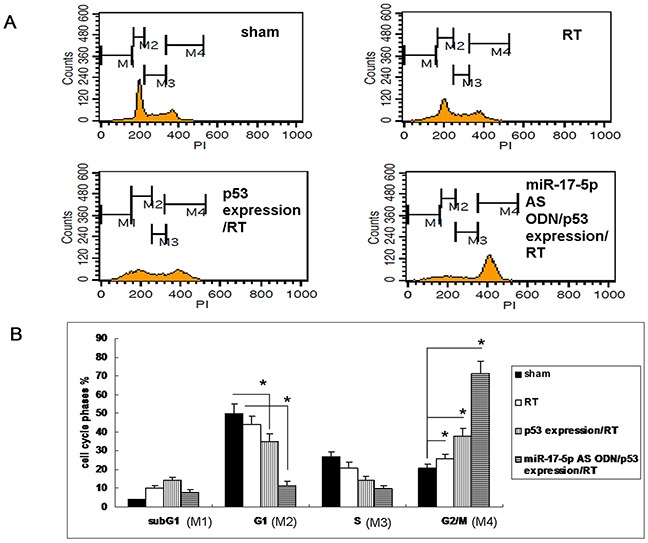
Overexpression of p53 protein-enhanced radiation-caused G2/M phase arrest in OC3 cells **A.** The OC3 cells without or with a p53-overexpressing clone or p53 over-expression clone that treated with miR-17-5p AS ODN were irradiated with 5 Gy; after 48 h, the cell cycle was determined through propidium iodide staining and flow cytometry. **B.** Quantitative results of the cell cycle phase fractions from (A). n = 3, *P < 0.05.

Many studies have revealed that when gamma radiation leads to a DNA damage response, DNA-damaging agents activate the p53 signaling pathway and cell cycle checkpoints to promote survival or apoptotic cell death [17–19]. Because miR-17-5p downregulated p-p53 (phosphorylated at S15, S46, and S392) in the OC3 cells, we further determined the effect of p53 on irradiated OC3 cells. We irradiated the OC3 cells without or with the p53-overexpressing clone and observed that 48 h after irradiating the cells with 5 Gy, G_2_/M phase arrest was significantly enhanced in p53-overexpressing OC3 cells (49.58 ± 3.5) compared with the wild-type OC3 cells (38.62 ± 2.4; Figures 4A and 4B). The effect of mir-17-5p AS ODN on cell cycle arrest in irradiated p53 expressing cells was also evaluated. While treated with mir-17-5p AS ODN to irradiated p53 expressing cells, the effect of p53 on cell cycle arrest was significantly enhanced (Figure 4). The results revealed that the effect of miR-17-5p on the expression of p53 contributed to the modulation of the radiosensitivity of irradiated OC3 cells.

### MicroRNA-17-5p antisense ODN therapy enhanced the radiosensitivity of OC3 tumor growth *in vivo*

**Figure 5:**
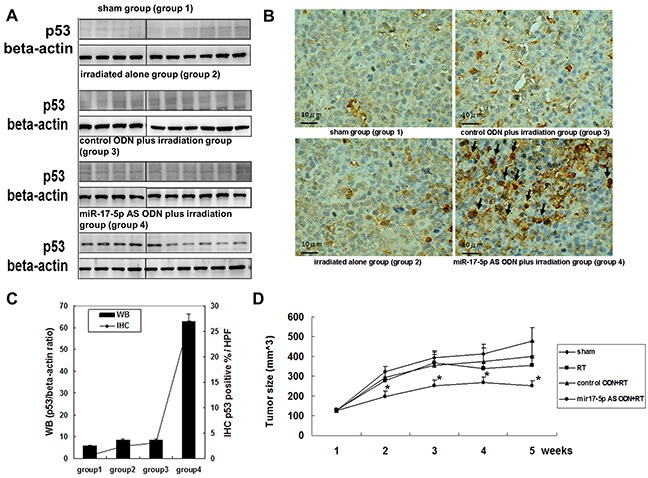
MiR-17-5p AS ODN therapy enhanced p53 expression and radiosensitivity of OC3 cell tumor growth in vivo OC3 cells were seeded in the legs of SCID mice (N = 40). After tumor formation on day 7, the mice were divided into four groups. The sham group (group 1); irradiation-alone group (group 2); control ODN plus irradiation group (group 3), and miR-17-5p AS ODN plus irradiation group (group 4). Both types of ODN were administrated through a tail vein injection (10 μg/mouse). The mice were treated with miR-17-5p AS ODN or control ODN on day 7; on day 8, the mice in groups 2–4 were processed for tumor irradiation (10 Gy). On day 9, all mice were processed for tumor biopsy. **A.** The tumor biopsy samples were used to determine p53 expression; beta-actin was used as the loading control. **B.** The tumor biopsy samples were used for immunohistochemically stained with anti-p53 antibody. **C.** The quantification of p53 protein expression in each group was done by calculating the ratio of relative intensity of p53 and beta-actin (immunoblotting) or the ratio of p53 positive rate in 5 high power field (HPF) per group (IHC). **D.** The tumor size of each mouse was estimated weekly. The tumor growth curve was generated. N=10/group. *P < 0.05.

To determine the in vivo therapeutic effect of miR-17-5p AS ODN, we used an OC3 xenograft model and SCID mice to determine the effect of miR-17-5p AS ODN on tumor irradiation. After tumor formation on day 7, 10 mice each were divided into the sham group (group 1), irradiation-alone group (group 2), control ODN plus irradiation group (group 3), and miR-17-5p AS ODN plus irradiation group (group 4). The mice were treated with miR-17-5p AS ODN or control ODN on day 7; on day 8, the mice in groups 2–4 were processed for tumor irradiation (10 Gy). On day 9, all mice were processed for tumor biopsy. Furthermore, the tumor biopsy samples were used to determine the expression of p53. There are no statistically significant changes on body weights among experimental groups. Western blotting revealed a considerably high expression of p53 in group 4 but not in groups 1–3 (Figure 5A). The IHC of p53 also revealed the p53 was abounding expressed in the miR-17-5p antisense ODN plus irradiation group (group 4) but not in other three groups (Figure 5B). The tumor size of each mouse was estimated weekly. The tumor growth curve revealed a higher inhibition of tumor growth in group 4 than in groups 1–3 (Figure 5C). The results revealed that the miR-17-5p-induced downregulation of p53 plays a critical role in OC3 radiosensitivity. These results indicated that miR-17-5p increased or decreased apoptosis-related proteins, namely p21, p53, TNF RI, FADD, cIAP-1, HIF-1α, and TRAIL R1 in the OC3 cells. Its effect on p53 protein expression, at least in part, contributed to the modulation of the radiosensitivity of the OC3 cells. These results also support that miR-17-5p AS ODN therapy may be a strategy for betel nut chewing-associated oral cancer.

## DISCUSSION

MiRs increase or decrease gene expressions at the post-transcriptional level by associating with RNA-inducing silencing complexes, which trigger RNA degradation and repress mRNA translation [10, 11]. MiR-17 encodes seven mature miRs, and several studies have revealed their involvement in cancer [20, 21]. Multiple studies have focused on investigating the role of in oral cancer [22, 23]. The overexpression of oncogenic miR may reduce the protein products of tumor-suppressor genes. Simultaneously, levels of oncogenic proteins may increase when tumor-suppressor miRs are inhibited. Detecting miRs involved in oral cancer could provide new strategies for treating and diagnosing oral cancer [24]. An antiapoptotic effect of the miR-17-5p–92 cluster was observed in cancer; in this study, we evaluated the function of the miR-17 polycistron in irradiated OC3 cells. We showed that irradiation induced miR-17-5p expression and played a role in suppressing apoptosis. As previously described, miRs can trigger RNA degradation and repress mRNA translation. Fontana et al. reported that in therapy-resistant neuroblastoma, an increased miR-17-5p expression causes the downregulation of p21 and the tumor suppressor gene Bcl-2-interacting mediator (BIM) of cell death, which could be antagonized by miR-17-5p AS ODN, representing a novel oncogenic pathway to explain neuroblastoma progression and its resistance to therapy [25]. In the present work, miR-17-5p downregulated the expression of p21, p-p53 (phosphorylated at S15, S46, and S392), TNF RI, and FADD, which might play roles in the radiosensitivity of irradiated tumor cells. Xie et al. reported that the phosphorylation of p53 could result in increased radiosensitivity and the inhibition of tumor reproduction [26]. Strigari et al. also demonstrated that p53-dependent signals might be responsible for the abscopal effect in RT to sterilize nonirradiated tumor cells through a proapoptotic pathway [27]. These outcomes were also compatible with our results that the overexpression of p53 enhanced the radiation-induced G2/M phase arrest of OC3 cells.

The p53 is modified post-translationally at multiple phosphorylation sites. DNA damage induces phosphorylation of p53 at S15, S20 and S37, reducing its interaction with the oncoprotein MDM2 [28]. The phosphorylation by cdk-activating kinase at S392 is increased in human tumors and has been reported to influence the growth suppressor function, DNA binding and transcriptional activation of p53 [29]. Phosphorylation of p53 at S46 regulates the ability of p53 to induce apoptosis [30]. Furthermore, Baierlein et al. reported a synergistic effect of the combined use of TNF-α and radiation on the radiosensitizing effect *in vitro* and *in vivo*, and combined-modality treatment may increase the therapeutic effectiveness of irradiation in bladder cancer. These results were similar to our findings on oral cancer [31]. Our findings suggest that the enhancement of TNF RI expression in tumors yield a more desirable radiation effect in betel nut chewing-related oral cancers. FADD, also called MORT1, is encoded by the *FADD* gene on the 11q13.3 region of chromosome 11 in humans [32]. In a study using proteomics analysis, the expression of various types of apoptosis-regulating proteins increased after radiation; these proteins involved the Fas antigen and a TNF-inducible protein after radiation. The results suggest that the expression of apoptosis-related proteins, such as FADD or TNF-inducible protein, play crucial roles in this radiosusceptibility [33]. In our study, effects of the miR-17-5p AS ODN on OC3 cells potentially enhanced the expression of apoptosis-related proteins to achieve radiosensitivity. Such a treatment might overcome the poor treatment outcomes in betel nut chewers with higher incidences of local recurrence and secondary primary tumors than nonchewers [3].

MR-17-5p upregulates the expression of cIAP1, HIF-1α, and TRAIL R1, which might play roles in the radioresistance of irradiated tumor cells. The overexpression of the antiapoptotic protein, cIAP1, caused by its genetic amplification was reported in certain cancers, such as hepatocellular carcinoma, esophageal squamous cell carcinoma, cervical cancer, and lung cancer, and this factor conferred resistance to chemotherapy and RT [34, 35]. Nagata et al. showed that cIAP overexpression contributed to therapeutic resistance and a poor prognosis in oral squamous cell carcinoma [36]. Imoto et al. demonstrated that some cervical cancer cell lines showed amplification and a consistent overexpression of cIAP1 as well as significant resistance to radiation-induced cell death compared with the cell lines showing no cIAP1 amplification [37]. However, no associated data are present on cIAP1 in irradiated betel nut chewing-related oral cancer cells. Our data might be the first to reveal that a high cIAP1 expression stimulated by miR-17-5p results in radiation resistance. Moreover, several studies have reported on HIF-1α overexpression-related radioresistance in head and neck cancers [38–40]. In the present study, we confirmed similar outcomes in irradiated betel nut chewing-related oral cancer cells. TRAIL, TNF-related apoptosis-inducing ligand receptor 1 (DR4), and TNF-related apoptosis-inducing ligand receptor 2 (DR5) can all be induced by chemotherapeutics or radiation, which can sensitize cancer cells to TRAIL. Thus, understanding the regulation of the TRAIL apoptosis pathway can help develop more selective TRAIL-based agents for treating human cancers [41]. Mapatumumab, a fully human agonistic monoclonal antibody to TRAIL R1 developed to induce apoptosis in cancer cells, might be promising, and it has minimal effects on normal cells [42]. Our study also revealed that miR-17-5p upregulates the expression of TRAIL R1, which might confer higher resistance to RT in betel nut chewing-related oral cancer. Moreover, we found that the effects of the miR-17-5p AS ODN on OC3 cells potentially reduced the expression of these radio-resistant proteins to overcome therapeutic resistance. Thus, we could use miR-17-5p AS ODN for multiple targets to enhance therapeutic effects.

Meanwhile, p21 (WAF1) plays a key role in the p53 pathway [43]. p21 is required in DNA damage-induced G_2_ arrest in normal human fibroblasts and cancer cells [44]. We demonstrated that miR-17-5p decreased the expression of the cyclin-dependent kinase inhibitor p21 in the OC3 cells following irradiation. Furthermore, antagomir-17-5p downregulated the expression of p21 and BIM in neuroblastomas through a mechanism that involved p21 and led to cell cycle blockade and the BIM activation of apoptosis, respectively [25]. Wang et al. studied the mechanism underlying p21 suppression by the miR-17 family and observed that c-Myc expression was associated with miR-17 family members. Meanwhile, c-Myc is a critical transcriptional repressor and not a transcriptional suppressor of p21 [45]. Wang et al. compared the expression of p21 mRNA in the nuclei and cytoplasm of stable c-Myc transfectants and control cells, and their results revealed that c-Myc repressed p21 at the post-transcriptional level. Furthermore, the stable overexpression of c-Myc elevated the expression of some members of the miR-17 family and of their primary transcripts. To confirm the association of c-Myc with the miR-17 family and p21, Wang et al. antagonized the expression of each miR-17 family member with a specific AS ODN in transfectants constitutively overexpressing c-Myc, in the presence of c-Myc, and showed that this treatment strongly restored p21 expression. These results suggest that c-Myc further repressed p21 expression at the post-transcriptional level in some members of the miR-17 family [45]. Leibowitz et al. compared animal survival, apoptosis, proliferation, cell cycle progression, DNA damage, and regeneration in the crypts of wild-type, p53-knockout, PUMA-knockout, p21-knockout, and p21/PUMA double-knockout mice in a whole-body irradiation model, in which intestinal damage was induced by radiation [34]. Those authors observed that a deficiency in p53 or p21 led to shortened survival but accelerated crypt regeneration associated with massive nonapoptotic cell death, including aberrant cell cycle progression, persistent DNA damage, considerable replication stress, and genome instability. Their study also revealed the major role of p21 in p53-dependent cell cycle checkpoints [19]. By using p21 AS ODN, we revealed that p21 plays a critical role in oral cancer cells. However, the radioresponse of betel nut-related oral cancer needs to be further investigated. Yang et al. investigated the radiosensitization effect of MLN4924, an SKP1, Cullins, and F-box protein E3 ubiquitin ligase substrate inhibitor [46], which is known to stimulate growth arrest, apoptosis, and the DNA-damage response; they observed that MLN4924 is a potent radiosensitizing target of breast cancer in the SK-BR-3 breast cancer cell line. Moreover, in MCF7 cells, p21 was overexpressed in the MLN4924 treatment plus radiation group but not in the single-treatment group. Yang et al. also evaluated the role of p21 by using small interfering RNA, and their results revealed that p21 knockdown partially inhibited MLN4924-induced G_2_/M arrest and radiosensitization. Our results also revealed the radiosensitization effect of p21 in OC3 cells. Moreover, Li et al. investigated cancerous miRs in LNCaP cells in response to RT by using a miR microarray assay and found an altered expression of mi-106b. Furthermore, those authors transfected the precursor miR-106b into LNCaP cells to investigate its role and observed that miR-106b expression suppressed radiation-induced p21 activation and induced G_2_/M arrest in response to radiation; this resulted in a transient decrease in radiation-induced growth inhibition [47]. In our study, we transfected the OC3 cells with miR-17-5p to verify its biological role; our results also showed G_2_/M arrest in response to radiation, which resulted in the inhibition of radiation-induced apoptosis. These results revealed that miR plays a rescuing effect in cancer RT. In our animal study, immunohistochemical analysis clearly revealed p21 expression and apoptosis in tumor tissues within 4 days after RT rather than 14 days later, when the molecular responses may have passed, potentially indicating that the change in tumor size was a net effect of RT. Clinical trials and mechanistic studies are urgently required to identify appropriate radiosensitizers for administering RT for oral squamous cell carcinoma [48–51]. Concurrent chemoradiation therapy has improved locoregional control and patient survival; however, locoregional failure remains a significant problem [52, 53]. Patients with individual head and neck cancers and advanced oral squamous cell carcinoma having tumors similar in sizes and stages may respond extremely differently to RT [54, 55].

AS ODNs have the ability to selectively block disease-causing genes, thereby inhibiting the production of disease-associated proteins. The specificity and application of AS ODN have been strongly validated in animal models for various disease targets [56–58]. In this study, we used miR-17-5p AS ODN in vivo; AS ODN may be a powerful tool for clinical use. According to our review of relevant literature, this is the first study to investigate and present preclinical data on the association and anticancer effects of miR-17-5p AS ODN in vivo and irradiated betel nut-related oral cancer. The preclinical animal data might be valuable for conducting further clinical trials for patients with betel nut chewing-related oral cancer because these patients have higher incidences of local recurrence, distant metastasis, and secondary primary cancers as well as poorer disease-specific and overall survival than do nonchewers [3]. Our finding from animal study revealed that microRNA-17-5p AS ODN therapy enhanced the radiosensitivity of OC-3 tumor growth, suggesting that miR-17-5p AS ODN therapy may be a strategy for betel nut chewing-associated oral cancer.

In conclusion, our results reveal that miR-17-5p plays a crucial role in the expression of apoptotic proteins, namely p21, p53, TNF RI, FADD, cIAP-1, HIF-1α, and TRAIL R1, in irradiated OC3 cells. These proteins play a critical role in the radiosensitivity of oral squamous cell carcinoma cells associated with betel nut chewing. These findings would be useful for designing therapeutic strategies, in which RT and miR-17-5p-based gene therapy can be co-administered.

## MATERIALS AND METHODS

### Cell culture

The OC3 cell line was obtained from the National Health Research Institutes, Miaoli County, Taiwan. The cells were cultivated in combined keratinocyte serum-free medium and Dulbecco's modified Eagle's medium (2:1, v/v, Invitrogen, USA) containing 10% fetal bovine serum.

### Radiation treatment

The flask was irradiated with different doses of radiation (0–5 Gy) by using a Cobalt-60 unit. The distance from the source to the skin was set to 80 cm at the bottom of the flask. The dose rate was approximately 1 Gy/min.

### Quantitative detection of miR-17-5p

The total RNA was extracted from irradiated OC3 cells by using the mirVana miRNA Isolation Kit (Ambion) according to manufacturer instructions. The total RNA was extracted from approximately 1 × 10^6^ cells and was followed by cDNA synthesis by using 1 μg RNA. Successive polymerase chain reaction (PCR) was performed using the QuantiMir-RT kit (System Biosciences, CA, USA). By using 1 μL of cDNA as the template, miR-17-5p was detected using a real-time PCR assay kit (Signosis, Inc. Sunnyvale, CA, USA). It facilitates oligoligation and SYBR green-based real-time PCR.

### MicroRNA-17-5p antisense oligonucleotides

A 5′-ACUACCUGCACUGUAAGCACUUUG-3′ 2′-O-methyl oligonucleotide (Dharmacon, USA) was used for miR-17-5p antisense oligonucleotide. A chemically synthesized 2′-O-methyl oligoribonucleotides, 5′-AAAACCUUUUGACCGACCGAGCG UGUU-3′, was used as the control ODN. The oligonucleotides were resuspended in DNase-free water to form solutions of 1 μM, mixed with an equal volume (10:1) of TransFast™ for 15 min, and incubated with the cells in a serum-free medium.

### p53 stable cells

The p53 ORF cDNA (Catalog # porf-hp53, InvivoGene, USA) was subcloned in pcDNA3 expression vector. The pcDNA-p53 expression vector, a constitutive expression vector carries full-length human p53 cDNA under the control by the CMV promoter/enhancer sequence, was transfected into theOC3 cell line to generate a p53 over-expression line with the TransFastTM transfection reagent (Promega). For selecting p53 over-expression line, culture medium was replaced by DMEM with 10% FBS and 600 μg/mL G418. Clones resistant to G418 were selected and expanded.

### Western blotting

The cells or tissue biopsy samples were homogenized in a protein lysis buffer. A 50-μg protein sample was separated through 10% sodium dodecyl sulfate polyacrylamide gel electrophoresis and transferred onto polyvinylidene difluoride membranes. After blocking, the membranes were immunoblotted with various primary antibodies, followed by incubation with the appropriate peroxidase-coupled secondary antibodies. Furthermore, the signal was visualized on an X-ray film after detecting with an enhanced chemiluminescent detection system. Antibodies to p21, p-p53 (phosphorylated at S15, S46, and S392), tumor necrosis factor receptor I (TNF RI), Fas-associated death domain protein (FADD), cellular inhibitor of apoptosis protein 1 (cIAP-1), hypoxia-inducible factor (HIF)-1α, and TNF-related apoptosis-inducing ligand receptor 1 (TRAIL R1) were purchased from R&D Systems.

### Cell cycle analysis

OC3 cells were harvested and fixed in 75% ethanol at −20°C for 1 h. After centrifugation (2000 r.p.m.) for 10 min at 4°C, the cell pellets were resuspended in 0.5 mL of buffer (0.5% Triton X-100/PBS and 0.05% RNase A), and incubated for 30 min at room temperature. Finally, 0.5 mL propidium iodide (PI) solution (50 μg/mL) was added and allowed to incubate on ice for 30 min. Fluorescence emitted from the PI-DNA complex was quantified after laser excitation of the fluorescent dye using FACSsor flow cytometry (Becton Dickinson). The cell cycle stages were analyzed by quantifying the DNA content with the Cell Quest software (Becton Dickinson).

### Human apoptosis protein array

A human apoptosis array (Proteome Profiler™ Ary009; R&D Systems) was used to analyze apoptosis-related protein profiles according to manufacturer instructions. In brief, the total cell lysates were first incubated with the array membrane overnight at 4°C, followed by incubation with a biotinylated detection antibody cocktail at room temperature for 1 h. A digital imaging system (Bio Pioneer Tech) was used to detect the chemiluminescent signals, which were further analyzed using the ImageJ program.

### OC3 cell xenograft model in SCID mice

Forty male severe combined immunodeficiency (SCID) mice (aged 8 weeks) were obtained from the Animal Center of the National Taiwan University College of Medicine. OC3 cells [1 × 10^6^ in a total volume of 0.02 mL of a serum-free medium containing 50% Matrigel (BD Biosciences, Bedford, MA, USA)] were directly injected into the right leg of the SCID mice. After tumor formation on day 7, mice were divided into four groups, including sham group (group 1), irradiated alone group (group 2), control ODN plus irradiation group (group 3), and miR-17-5p antisense ODN plus irradiation group (group 4). Both types of ODN were administrated by tail vein injection (10 μg/mouse). Mice were treated with miR-17-5p antisense ODN or control ODN on day 7 and 8, mice in groups 2 to 4 were processed for tumor irradiation (10 Gy). On day 9, all mice were processed for tumor biopsy: (A) The tumor biopsy samples were used to determine the expression of p53, beta-actin was used as loading control; (B) The tumor biopsy samples were used for immunohistochemically stained with anti-p53 antibody; (C) The tumor size of each mouse was estimated weekly. The tumor growth curve was generated. All experimental procedures using these mice were performed in accordance with the protocols approved by the Institutional Animal Care and Use Committee of National Taiwan University.

### Immunohistochemical assay

The biopsies were immediately fixed in a buffered formalin(4%) solution. Standard paraffin blocks were prepared and 5-μm sections were deparaffinized in xylene and rehydrated through graded concentrations of alcohol to distilled water. After antigen retrieval by heat treatment in a 0.1 M citrate buffer at pH 6.0, endogenous peroxidase activity was blocked using a 3% H_2_O_2_ solution. Slides were then incubated for 30 min in 2.5% normal donkey serum or goat serum. Afterward, the slides were incubated overnight at 4°C either with p53 antibody (1:20; sc-1311-R(C-19); Santa Cruz Biotechnology, Santa Cruz, CA), and following incubated with secondary antibodies according to the manufacturer's instructions. Finally, antibody binding was detected using the avidin-biotin-peroxidase method. Reaction products were developed using 3′, 5′-diaminobenzidine (Dako, Glostrup, Denmark) as a substrate for peroxidase. Sections were counterstained with Mayer's hematoxylin. All washes were performed using a phosphate-buffered saline solution (pH 7.4). Nuclear staining was considered as positive for p53.

### Statistical analysis

Data are presented as mean ± standard deviation for the indicated number of mice at each time point and in each group. The Student *t* test was used for statistical analysis, and P < 0.05 was considered significant.
